# HiComet: a high-throughput comet analysis tool for large-scale DNA damage assessment

**DOI:** 10.1186/s12859-018-2015-7

**Published:** 2018-02-19

**Authors:** Taehoon Lee, Sungmin Lee, Woo Young Sim, Yu Mi Jung, Sunmi Han, Joong-Ho Won, Hyeyoung Min, Sungroh Yoon

**Affiliations:** 1 Department of Electrical and Computer Engineering, Seoul National University, Seoul, 08826 Korea; 2 R&D Center, Wearable Healthcare, Gyeonggi-do, 16954 Korea; 3 Research Division, NanoEnTek, Seoul, 08389 Korea; 4 Department of Statistics, Seoul National University, Seoul, 08826 Korea; 5 College of Pharmachy, Chung-Ang University, Seoul, 06974 Korea; 6 Bioinformatics Institute, Seoul National University, Seoul, 08826 Korea

**Keywords:** Comet assay, Single cell electrophoresis, Image processing, Segmentation

## Abstract

**Background:**

DNA damage causes aging, cancer, and other serious diseases. The comet assay can detect multiple types of DNA lesions with high sensitivity, and it has been widely applied. Although comet assay platforms have improved the limited throughput and reproducibility of traditional assays in recent times, analyzing large quantities of comet data often requires a tremendous human effort. To overcome this challenge, we proposed HiComet, a computational tool that can rapidly recognize and characterize a large number of comets, using little user intervention.

**Results:**

We tested HiComet with real data from 35 high-throughput comet assay experiments, with over 700 comets in total. The proposed method provided unprecedented levels of performance as an automated comet recognition tool in terms of robustness (measured by precision and recall) and throughput.

**Conclusions:**

HiComet is an automated tool for high-throughput comet-assay analysis and could significantly facilitate characterization of individual comets by accelerating its most rate-limiting step. An online implementation of HiComet is freely available at https://github.com/taehoonlee/HiComet/.

## Background

DNA damage is known to be a major cause of cancer and many aging-related diseases [1]. The comet assay, also known as the single-cell gel electrophoresis, allows us to directly visualize DNA damage at the individual cell level [2]. Compared to other assays for DNA damage assessment, the comet assay is advantageous in terms of cost, sensitivity, and the ability to show multiple DNA lesions simultaneously [3]; it has been widely used in a variety of applications, including screening for breast cancer [4] and risk prediction for bladder cancer [5].

**Fig. 1:**
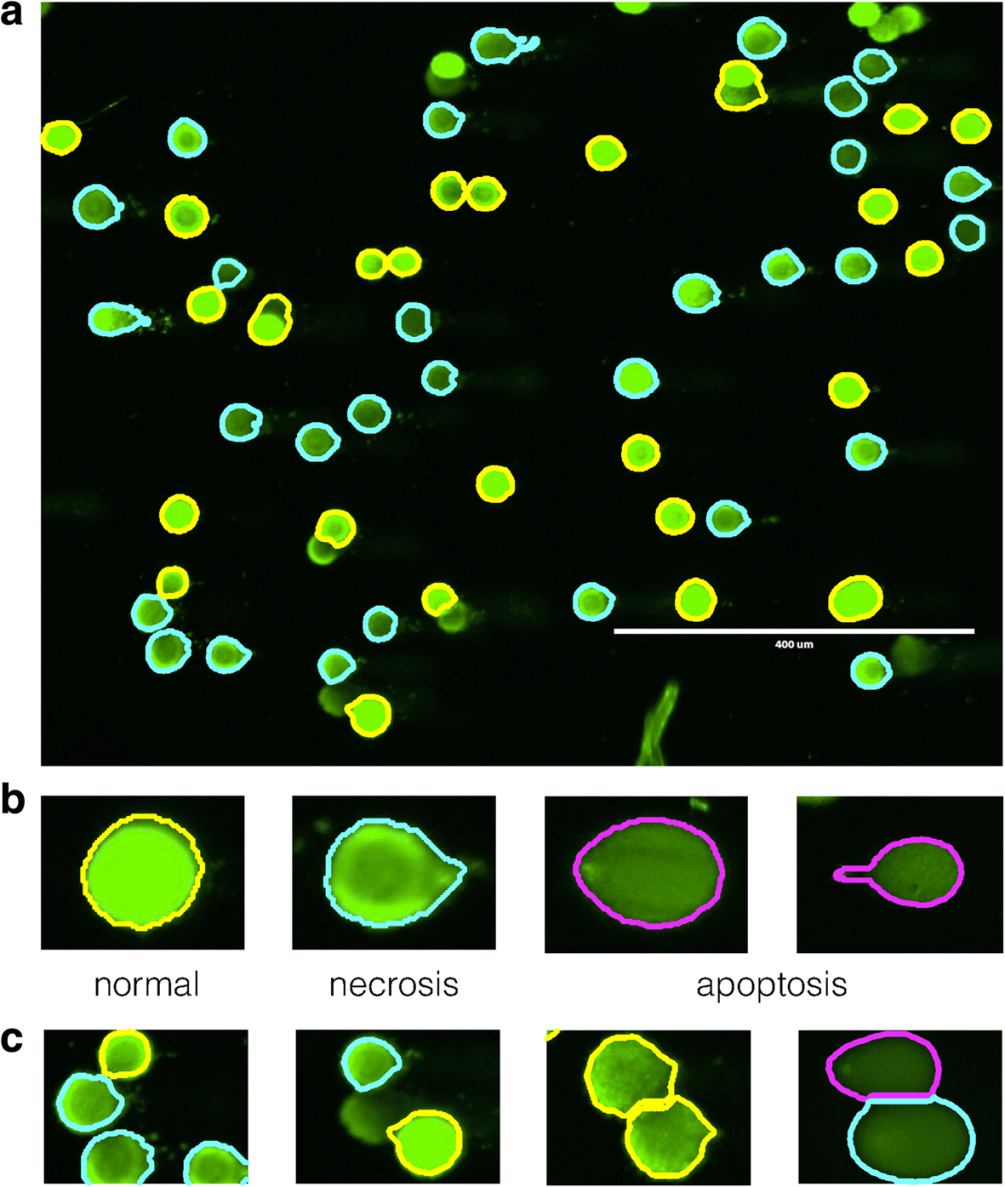
Comet assay: **a** A high-throughput comet assay produces an output image with multiple comets. **b** Comets are classified into different types according to their shape [1]. **c** HiComet can automatically identify and differentiate between overlapped comets

**Fig. 2:**
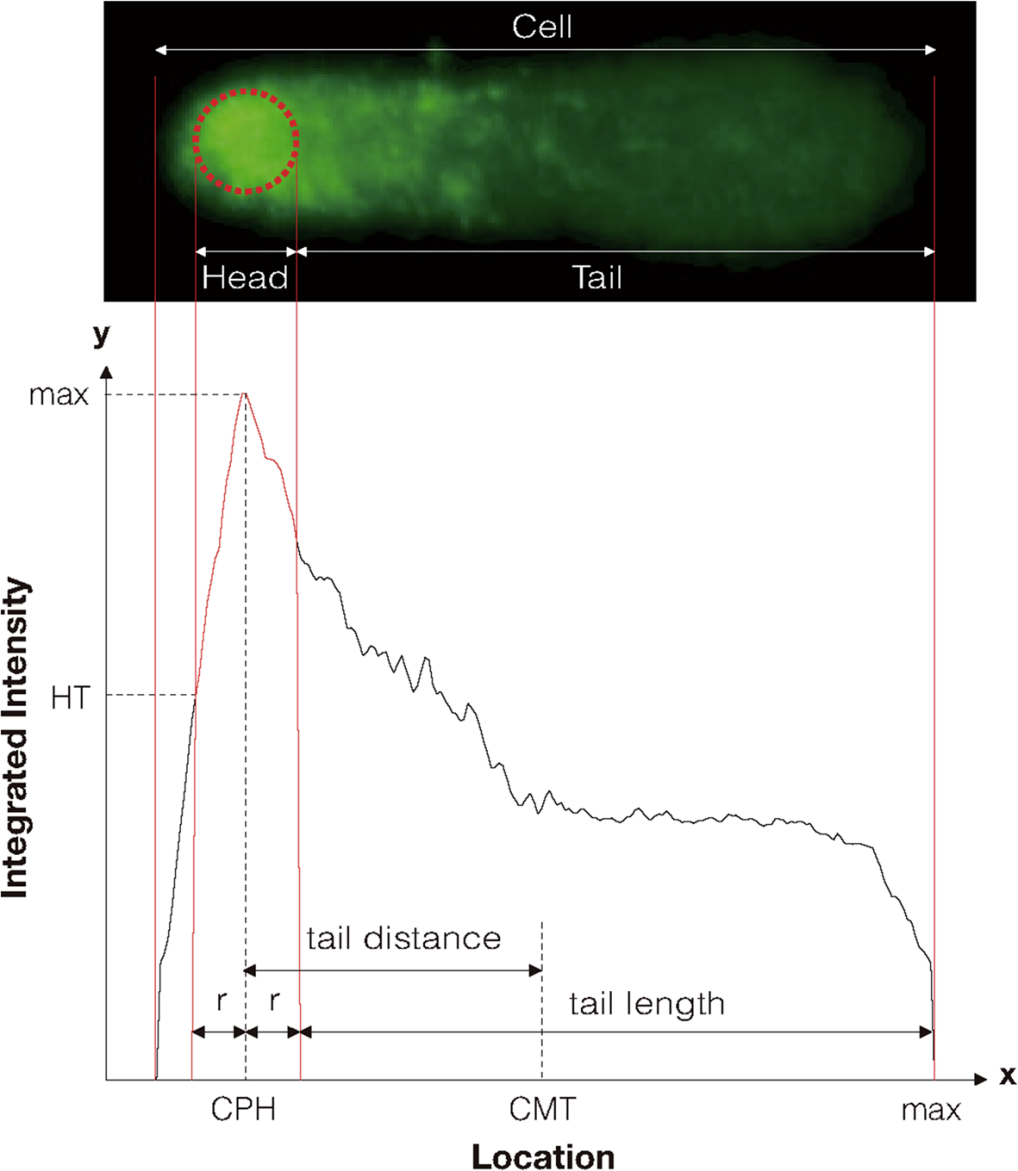
Definition of parameters that characterize a comet. Abbreviations: CPH, center position of head; CMT, center of mass of tail; HT, head threshold; *r*, head radius

In essence, the comet assay has the following steps [2, 3]. Cells treated with a DNA damaging agent (e.g., irradiation) are lysed and loaded onto an agarose gel. An electric field is applied to pull the negatively charged DNA from the nucleus. The DNA is stained with a fluorescent dye, and the resulting images appear as ‘comets’ (Fig. 1). Damaged DNA fragments migrate farther than normal ones; the relaxed loops and fragments form the tail of the comet, whereas the head comprises tightly packed chromatin (Fig. 2). As the dose of the DNA damaging agent increases, the comet head grows dimmer and the tail grows longer and brighter.

Traditional comet assays often have low throughput, limited reproducibility, and time-consuming and error-prone analysis steps. To overcome these issues, new comet assay platforms have been proposed [3, 6–8]. Although these platforms have various new aspects, the basic principle behind the analysis has remained unchanged: identification and characterization of individual comets. Due to the overlap of comets and debris, most of the existing analysis programs require laborious manual identification of comets from the fluorescent images. For high-throughput experiments that give a large number of comets, this comet recognition step forms a major bottleneck for the whole analysis pipeline, and there is a clear need for automation.

Pioneering attempts to automate high-throughput comet analysis have had limited success. A method to perform automated imaging and analysis has been proposed [3], but it is limited to microwell-array based comet assays that have highly regular structures with predetermined comet locations [3]. A commercial program called CometScore (TriTek Corp., Sumerduck, VA) can handle comet images in arbitrary configurations; however, it is only semi-automated, and the boundary of comets need to be specified for its automated characterization.

In this paper, we proposed HiComet, a computational tool to facilitate the analysis of high-throughput comet-assay data. Given a noisy image with a number of arbitrarily placed comets, HiComet can recognize and characterize normal and damaged comets in a fully automated manner, handling debris and overlaps between comets. Identifying individual comets in the input image is associated with the problem of image segmentation [9], but existing image segmentation techniques tend to be unsatisfactory when applied to this problem. HiComet utilizes a suite of new algorithms tailored for recognizing and characterizing comets in a rapid and robust manner.

### Understanding comet images

Figure 2 shows a typical comet image and the parameters for characterizing a comet. Each comet image shows the DNA damage in a single cell and consists of two major parts, namely the head and tail. The intensity and arrangement of the pixels convey important information. As damage to the DNA increases, the head becomes dimmer and the tail grows longer and brighter [10]. For instance, the three images in Fig. 1b show comets representing the cells in normal, necrosis, and apoptosis states, respectively, from left to right.

By considering intensity as mass, we can convert definitions from classical physics (such as center of mass and moment of inertia) into parameters that characterize comets. The illustration in the bottom pane of Fig. 2 introduces the key parameters of a comet image. The *x* axis corresponds to the horizontal location of pixels, and the *y* axis indicates the intensity across vertical direction.

The outline of a comet head is modeled on a circle. The *center position of head* (CPH) refers to the location of the head center in the *x* axis, and is defined as the peak position in the intensity curve. A user-specified parameter, called the *head threshold* (HT), specifies a fraction of the maximum intensity, and is used to define the head size. Thus, we define the radius *r* of the head as the distance between the CPH and the location with integrated intensity corresponding to HT [11].

The tail stretches from the right end of the head to the location where the intensity reaches zero, and the distance between these two points defines the tail length. We can compute the *center of mass of tail* (CMT) on the *x* axis, and the tail distance is defined as the difference between the CMT and the CPH.

It is customary to assume that the total amount of DNA in a comet is proportional to the sum of intensity values of all the pixels representing the comet [11]. That is,

1 $$\begin{array}{*{20}l} \textrm{DNA}&=\sum\limits_{x \in \text{comet}} I(x) \end{array} $$

2 $$\begin{array}{*{20}l} \textrm{TDNA}&=\frac{1}{\textrm{DNA}}\sum\limits_{x \in \text{tail}} I(x) \end{array} $$

where DNA and TDNA represent the amount of DNA in the cell (represented by the entire comet) and in the tail, respectively, and *I*(*x*) the intensity of pixels at *x*.

### Assessing DNA damage from tail shape

Multiple methods have been proposed to quantify the degree of DNA damage from the tail image. The simplest one is to consider the amount of DNA in the tail and the tail length together, which defines the (tail) *extent moment* [12]:

3 $$\begin{array}{*{20}l} \textrm{extent moment} = \textrm{TDNA} \times \textrm{tail length} \end{array} $$

Note that higher the extent moment, higher the DNA damage. One limitation of using the extent moment is the difficulty in differentiating comets with identical TDNA and tail length, but of different shapes.

To overcome this limitation, Olive et al. [10] had proposed the (tail) *Olive moment*, which is defined as follows:

4 $$\begin{array}{*{20}l} \textrm{Olive moment} = \textrm{TDNA} \times \textrm{tail distance} \end{array} $$

which involves the CMT in the calculation by using the tail distance instead of the tail length.

Furthermore, we can consider the distribution of pixels in the tail by using the *moment of inertia* of the tail [11], which is defined as

5 $$\begin{array}{*{20}l} \textrm{moment of intertia} = \frac{1}{\textrm{DNA}} \sum\limits_{x \in \text{tail}} I(x) \times (\textrm{CPH}-x)^{2} \end{array} $$

where the last term represents the squared distance between the CPH and each pixel in the tail.

### Image segementation

The problem of *image segmentation* concerns the recognition and extraction of objects embedded in a background image. In this study, image segmentation techniques play a key role in the fully automated recognition and characterization of comets, as will be elaborated.

**Fig. 3:**
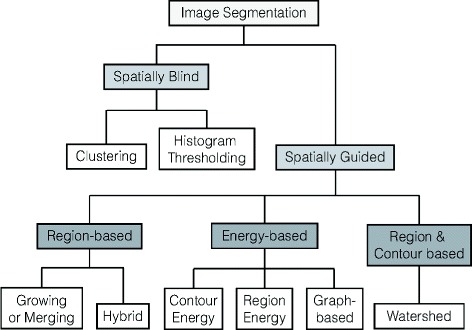
Taxonomy of image segmentation methods [9]

Figure 3 shows the taxonomy of existing image segmentation techniques [9]. Broadly, there are two approaches to the image segmentation problem, namely the spatially blind approach and the spatially guided approach. The approach is chosen based on the need for additional information (such as the gradient of regions and edges in the image).

In this study, we employed both types of image segmentation approaches in turn: the spatially blind method for initial comet identification and the spatially guided technique for further processing the overlapping comet images. We aimed to fully automate the comet image processing, and spatially blind methods are better suited for the initial comet identification. Based on the information obtained from it, we performed out spatially guided processing of overlapping comet images.

In the domain of spatially blind methods, we utilized a histogram-thresholding technique for comet identification, as described in “Methods” section. Clustering-based techniques would be an ideal alternative due to the simplicity and ease of implementation, but it is often difficult to determine the right number of clusters to yield satisfactory results. We compared the performance of the tested alternatives in “Results and discussion” section.

For the spatially guided approach, we employed the watershed method [13], an elegant segmentation tool based on morphological shapes. In this method, a gray-level image is considered a topographic relief, and the intensity of a pixel corresponds to the elevation at the pixel point. The contour of an object in the image is called the *watershed* and can be determined as the limits of the catchment basins of water drops flowing on the topographic relief. Our approach is elaborated in “Methods” section.

## Methods

**Fig. 4:**
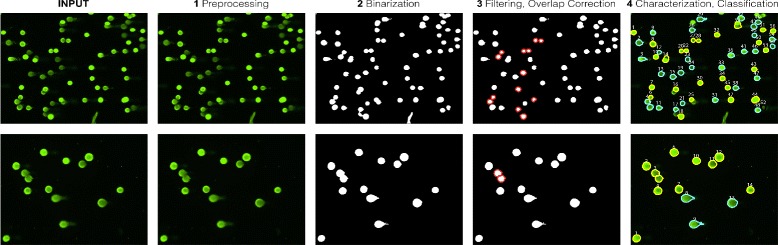
Overview of proposed methodology for HiComet

Figure 4 shows the overall proposed methodology for HiComet, which consists of four major steps: preprocessing, binarization, filtering, and characterization. The input is an image (in 8-bit RGB format) containing multiple comets obtained from a high-throughput comet-assay experiment with no limitations on comet location or quantity. HiComet does not assume a specific configuration of comet locations, which is an important advantage over existing software tools. Throughout the four-step pipeline, HiComet characterizes each comet, and extracts their parameters such as intensity profiles and tail moments. The output from HiComet comprises the images of individual comets and their characterization data.

The four steps of HiComet are elaborated in this section.

### Step 1: Preprocessing

This step involves a smoothing procedure, including median filtering or moving-average filtering. A moving window is created, and every pixel in the window is replaced with the median or average values within the window. This blurring operation reduces noise, thus facilitating downstream processing. In particular, the blurring operation was found to improve the accuracy of the thresholding for the binarization and to decrease erroneous dissection of the head and tail.

### Step 2: Binarization

This step distinguishes and separates objects from the background. The pixel intensity of the comet assay images corresponds to the density of the cell fragments. So, we used a simple thresholding method. One of the well-known thresholding methods is Otsu’s method. The algorithm performs minimization of the within-class variance $\sigma ^{2}_{W}$, or alternatively, maximization of the between-class variance $\sigma ^{2}_{B}$, defined by the following equations [14]:

$$\begin{array}{@{}rcl@{}} \sigma^{2}_{W} & = &\omega_{0} \sigma^{2}_{0} + \omega_{1} \sigma^{2}_{1}, \\ \sigma^{2}_{B} & = &\omega_{0} \omega_{1} (\mu_{1} - \mu_{2})^{2},  \end{array} $$

where *ω*_*i*_, *μ*_*i*_, and *σ*_*i*_ denote the probability of occurrence, the mean intensity, and the variance of intensity values for class *i*, respectively. Although Otsu’s method has been used for high contrast images [15], it was not suitable for comet assay images due to the variance of intensities in the comet pixels.

**Fig. 5:**
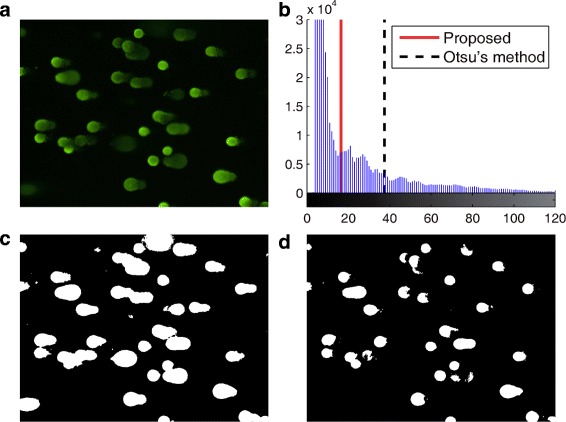
Binarization example: **a** original image **b** intensity histogram of grayscale image **c** proposed binarization, and **d** Otsu’s binarization

As shown in Fig. 5a, comet pixels have a wide intensity range. Therefore, Otsu’s method could not detect faint areas, which are mostly abnormal cells that should ideally be detected (see Fig. 5d). To overcome this problem of under-segmentation, we generated a gray level histogram and sought the first valley point, as indicated in Fig. 5b. Gray level histograms of comet assay images always have the first peak at background intensity. While Otsu’s method always results in a high threshold because it minimizes the variance of intensities for comets, the first valley is placed somewhere between the background and Otsu’s threshold. Thus, relying on the first valley of a histogram gives the effect of using an adaptive threshold to distinguish comet and background pixels, as illustrated in Fig. 5b.

Because detecting the first valley might cause over-segmentation (see Fig. 5c), the filtering described in the next subsection will focus on removing false positives, to ensure elaborate contours for individual comets. After this preliminary segmentation, HiComet performs grouping of adjacent pixels based on 8-pixel connectivity and labels the identified comet candidates. This step completes the first round of comet identification.

### Step 3: Filtering and overlap correction

From the comets identified in the previous step, HiComet discards the “incomplete” ones from subsequent analyses. The aim of the previous step was to detect all possible comet areas, although false comet areas may also be included. In this step, HiComet first removes the comets lying on the boundaries of the input image, because most of them have invalid shapes. HiComet then identifies overly small groups of pixels, namely those with number of pixels lesser than a threshold (e.g., 0.1% of the total pixels in the input image). Such small groups are not removed immediately, but are merged into the closest comets. According to our results, these small groups influence the shapes of certain types of comets (e.g., the apoptosis type; Fig. 1b).

**Fig. 6:**
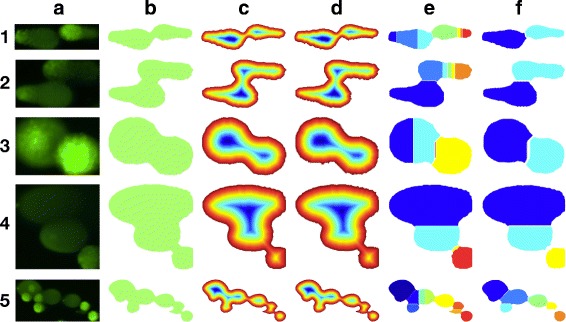
Overlap correction process (best viewed in color): **a** original image, **b** initial mask, **c** distance transform of **b**, **d** wavelet transform of **c**, **e** watershed transform of **d**, and **f** merging and filtering

After the filtering step, we detected overlapping comets and corrected them. Figure 6 shows the proposed overlap correction process. About five overlap examples are shown in Fig. 6a; we had obtained the initial masks depicted in Fig. 6b in the previous step. To identify the number of morphological shapes in these masks, we performed the watershed transform, followed by the distance transform. The watershed operator can detect multiple overlapping shapes in the ideal cases of smooth contour. However, in reality, the overlapping comets have noisy borders, and the individual comets among them have irregular shapes.

In order to address the robustness of the watershed, we applied the wavelet transform after the distance transform. The distance transform works by generating a topographic relief (see Fig. 6c). Each pixel has an altitude, which is calculated as the distance to the nearest boundary pixel in a binary image. The topographic relief may have many shallow holes, which cause over-segmentation during the watershed transform. Therefore, we utilized the wavelet transform as a smoothing filter for the topographic map, as shown in Fig. 6d.

We then obtained candidates of individual comets in the original binary image using the watershed transform (see Fig. 6e). However, over-partitioned chunks may still exist. This problem can be solved easily by merging horizontally divided areas into one segment, because the horizontal divisions arise from irregular contours of one shape. Figure 6f shows the results after a series of processes, but before the final filtering step.

**Fig. 7:**
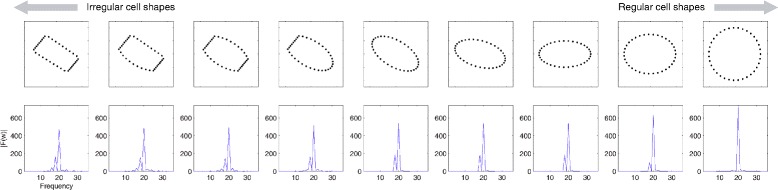
Fourier descriptors on 9 different shapes. Cells are generally elliptical, in which low-order frequencies are dominant

After the horizontal merging, the validity of each chunk must be verified. We assumed that all the cells were elliptical, and exploited the Fourier shape descriptor [16] to decide the roundness of each cell. Figure 7 shows the characteristics of the Fourier descriptor with 9 different shapes. An object on the frequency domain with the Fourier transform of 2D coordinates of contour points was visible. Low-order frequencies are more dominant, as cells were assumed to be elliptical. Based on this observation, we established two criteria to decide the validity of each chunk. First, we checked if the absolute sum of amplitudes of the two lowest frequencies was greater than 70% of the absolute sum of all the frequencies. Second, we discarded a chunk if its area over the area of the initial mask was lower than 3%. Thus, we obtained the partitioned segments shown in Fig. 6f.

### Step 4: Characterization and classification

**Fig. 8:**
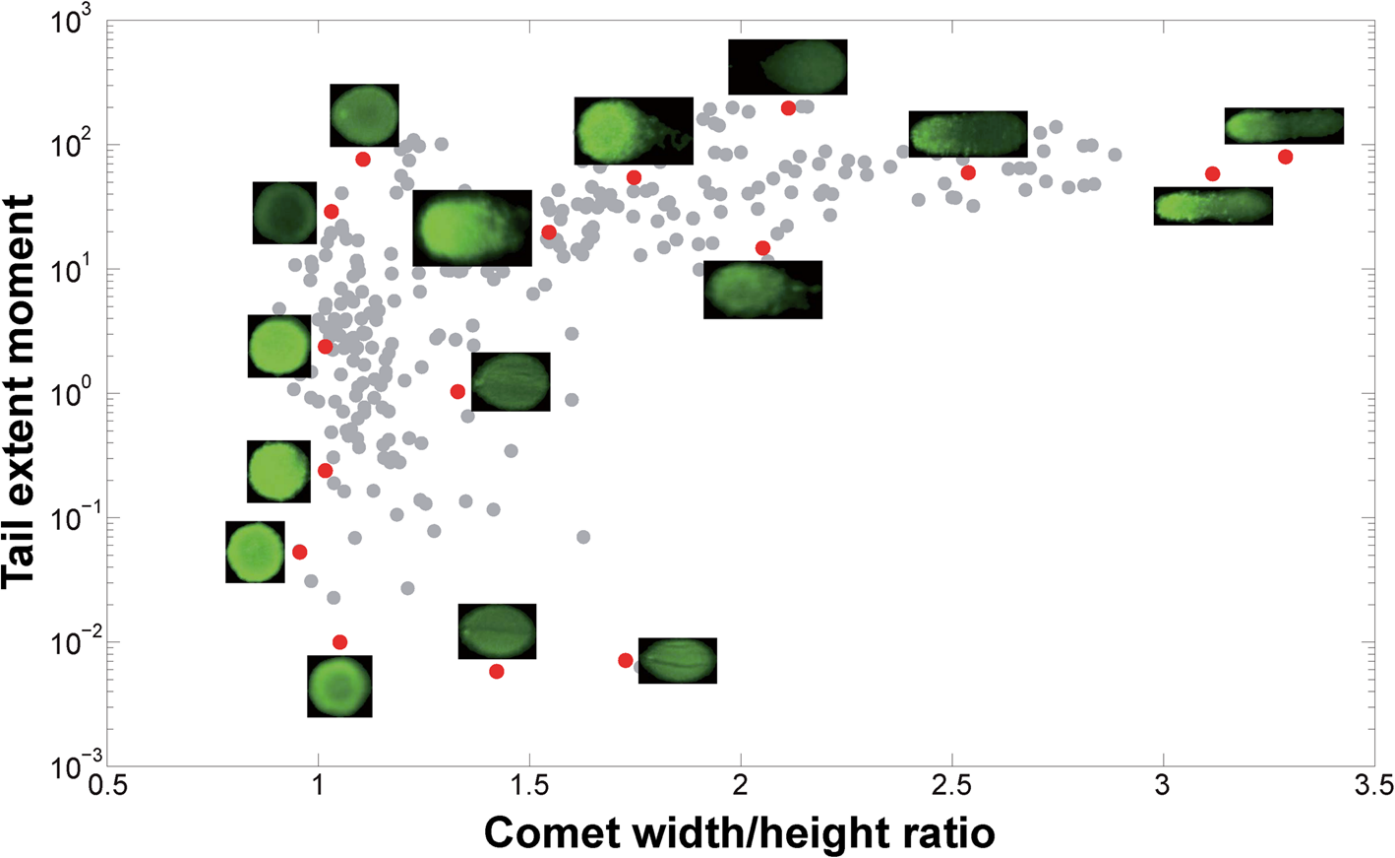
Characterization of comets. The *x*-axis and *y*-axis represent the width/height ratio and the tail extent moment, respectively. This plot characterizes 300 comets sampled from the 35 test images (Table 1), each of which appears as a blue dot; some dots are accompanied by comet images for visual inspection

The final step is to characterize each comet to find its parameters, including size, heads, tails, and tail moments. The tail moment in particular plays a key role in assessing the degree of DNA damage of a cell. HiComet reports three types of tail moments: the extent and Olive moments, and the moment of inertia, as mentioned in “Background” section. Figure 8 shows an example of comet characterization, with the distribution of some 300 comets in terms of the extent moment and the width/height ratio.

**Table 1:** Details of the 35 test images used

Img ID	N^*†*^	TP	FP	FN	Precision	Recall	F1-score	AUC
1	19	19	1	0	0.95	1.00	0.97	0.97
2	27	27	3	0	0.90	1.00	0.95	0.78
3	22	19	1	3	0.95	0.86	0.90	0.82
4	18	14	3	4	0.82	0.78	0.80	0.75
5	24	23	0	1	1.00	0.96	0.98	0.85
6	14	14	0	0	1.00	1.00	1.00	0.93
7	18	16	2	2	0.89	0.89	0.89	0.72
8	10	10	0	0	1.00	1.00	1.00	1.00
9	11	10	2	1	0.83	0.91	0.87	0.72
10	15	15	1	0	0.94	1.00	0.97	0.84
11	12	12	1	0	0.92	1.00	0.96	0.96
12	40	36	4	4	0.90	0.90	0.90	0.90
13	45	38	5	7	0.88	0.84	0.86	0.83
14	8	8	0	0	1.00	1.00	1.00	0.88
15	14	11	2	3	0.85	0.79	0.81	0.82
16	13	12	1	1	0.92	0.92	0.92	0.92
17	17	16	1	1	0.94	0.94	0.94	0.83
18	21	17	0	4	1.00	0.81	0.89	0.79
19	24	20	2	4	0.91	0.83	0.87	0.77
20	21	16	1	5	0.94	0.76	0.84	0.86
21	42	41	2	1	0.95	0.98	0.96	0.94
22	46	37	5	9	0.88	0.80	0.84	0.78
23	44	39	6	5	0.87	0.89	0.88	0.84
24	56	54	1	2	0.98	0.96	0.97	0.88
25	12	11	0	1	1.00	0.92	0.96	0.92
26	15	15	1	0	0.94	1.00	0.97	0.84
27	9	9	2	0	0.82	1.00	0.90	0.73
28	10	10	0	0	1.00	1.00	1.00	0.70
29	13	13	0	0	1.00	1.00	1.00	0.77
30	11	10	0	1	1.00	0.91	0.95	0.95
31	8	7	0	1	1.00	0.88	0.93	0.81
32	9	9	0	0	1.00	1.00	1.00	1.00
33	13	13	0	0	1.00	1.00	1.00	1.00
34	8	6	1	2	0.86	0.75	0.80	0.79
35	13	12	3	1	0.80	0.92	0.86	0.77
Total	702							
Average	20.06				0.93	0.92	0.92	0.85

N^*†*^ denotes the number of comets

After the characterization, we extracted the *histogram of oriented gradients* (HOG) [17] features from each cell image. HOG is a feature descriptor used in computer vision and image processing for object detection. The technique counts the number of occurrences of gradient orientation in localized portions of an image. We compared 4 classifiers with the HOG features, and the experimental results are described in the next section.

### Test data preparation

To evaluate the performance of HiComet, we tested it with 35 golden data sets verified by domain experts. Each data set was based on images from a micro comet-assay system (PICASSo, currently under development, NanoEnTek Inc., Korea). The system consists of a gel-electrophoresis microchamber and parallel multi-microchannels, which enables the loading of a low-melting point agarose (LMA) gel mixed with single cells. In these experiments, Jurkat cells were exposed to a toxic material (20 mM hydrogen peroxide) for 10 min and loaded into the multi-microchannels. After electrophoresis and nucleic acid staining with SYBR green, fluorescent images were captured with a microscope (EVOS, AMG Inc., USA). Three domain experts visually identified the comets from each of the 35 images, reporting 8–56 comets per image (20.03 on average and 702 in total). The throughput for processing these test images was over 1000 comets per minute. Details of the sample images are listed in Table 1.

## Results and discussion

### Effective image segmentation

We evaluated the binarization for the 35 test images with a region-based measure. The segmentation method labeled each pixel of the image with a binary value, identifying whether it was a comet pixel or not. We regarded the binarization of a comet assay image as a binary vector, and calculated true positives (TPs), false positives (FPs), true negatives (TNs), and false negatives (FNs). TP (TN) refers to a comet (background) pixel that is correctly binarized as a comet (background). FP is a background pixel that is incorrectly binarized as a comet. For each image, we calculated precisions and recalls, with the following equation:

$$\begin{array}{*{20}l} \text{precision}& = \text{TP} / (\text{TP}+\text{FP}),\\ \text{recall}&= \text{TP} / (\text{TP}+\text{FN}). \end{array} $$

**Fig. 9:**
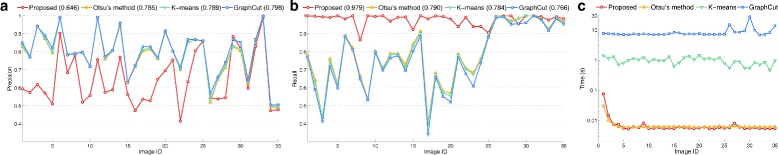
Comparison of binarization performances in terms of **a** precision, **b** recall, and **c** runtime

We compared the proposed binarization with three alternatives: Otsu’s method [14], K-means [18], and GraphCut [19]. Figure 9 shows the precision, recall, and runtime of these four methods on all the test images. On average, HiComet resulted in 15.8% lower precision values than GraphCut (0.646 versus 0.798). However, HiComet outperformed the three alternatives in terms of recall, yielding up to 21.3% higher average recall value than GraphCut.

In the binarization, we aimed to perform preliminary segmentation for the following enhancement procedure. By minimizing FNs, which are incorrectly segmented actual positives, we can preserve the true comet areas in the first round and concentrate on minimizing of FPs in the next filtering step. The proposed thresholding not only identified actual comet pixels successfully (0.979 recall on average) but also achieved this in just 0.1 s. Because the boundary of the comets were smeared and blurry due to the DNA fragments, the three alternatives that focused on detecting objects with crisp boundaries missed many fragmented pixels around the main body. This is understandable, given that these methods were designed for general images, in which clean-cut image segmentation is required.

### Fully automated identification of comets

By comparing the number of TPs, FPs, and FNs in each test image analyzed using by HiComet and the reference identification procedures. In order to count the correctly detected cells, we used a *centroid-based* measure instead of the *region-based* measure described in the previous subsection. A cell was considered TP if its centroid was located within a range of 15 pixels (=12*μ*m) from the centroid of a ground truth cell. Based on these numbers, we calculated the precisions, recalls, and F1-scores for each image, as listed in Table 1. The F1-score is defined as the harmonic mean of a precision and a recall.

Through the filtering and overlap correction steps (Step 3), HiComet raised the average precision and recall values from 0.74 and 0.73 to 0.93 and 0.92, respectively. For GraphCut, the average precision and recall were 0.75 and 0.66, despite applying the correction procedure. The four methods described in the previous subsection were ranked according to the centroid-based F1-score, and the result showed that the adaptive thresholding method was the best (0.92), followed by Otsu’s method (0.77), K-means (0.76), and GraphCut (0.71). Even if we utilized the three alternatives without the filtering, we could obtain up to 0.6 average F1-scores. For comet assay images with cloudy object boundaries, we believe that the proposed thresholding and correction schemes should be used together to detect all comets, including blurred and noisy ones.

### Quantification and classification of DNA damage states

Based on the HOG feature descriptor, we utilized four classifiers for discriminating between three types: normal, necrosis, and apoptosis. The classifiers we tested were support vector machine (SVM) [20], neural networks (NN) [21], AdaBoost [22], and the classification and regression tree (CART) [23]. Details of the individual classifiers are described as follows.

For SVM, we trained three binary classifiers (normal vs. necrosis, normal vs. apoptosis, and necrosis vs. apoptosis) and aggregated them into a decision tree: a traditional approach for multiclass SVM. We tried four well-known kernels (linear, polynomial, sigmoid, and radial basis function), among which the linear kernel showed the best performance. For NN, we constructed only input and output layers, composed of three nodes with softmax regression. For AdaBoost, we selected a decision tree as a template classifier and exploited 200 trees. For CART, the maximum pruning level was set to 10.

We assumed that apoptosis always has a nucleus in the left-half area (see Fig. 11b), and therefore collected two HOG features with overlapping 8×8 grids from all the resized (50×50) and left-half images (25×50). In order to discriminate between the horizontally long types (e.g., necrosis; see Fig. 11d), the box-ratio, which is defined as the width divided by height of a cell image, was used. Table 2 shows the classification performance for the four classifiers and the four feature sets. All the numbers depicted are averages from 10-fold cross-validation. The CART showed overfitted predictive performances; the linear SVM resulted in the highest test accuracy (0.904) with all the features, among the 16 possible combinations.

**Table 2:** Classification performances

Feature set	# of features	Training accuracy				Test accuracy			
		SVM	NN	AdaBoost	CART	SVM	NN	AdaBoost	CART
HOG 8 × 8 from	360	0.921	0.947	0.818	0.968	0.865	0.833	0.778	0.726
left-half									
HOG 8 ×8 from	361	0.935	0.956	0.867	0.963	0.877	0.869	0.836	0.752
left-half & boxratio									
HOG 8 ×8	900	0.985	0.984	0.833	0.972	0.873	0.864	0.773	0.749
HOG 8 ×8 & boxratio	901	0.989	0.987	0.867	0.971	0.904	0.872	0.823	0.786

After the classification, HiComet could identify around 90% of non-overlapped cells in one comet assay image. In order to evaluate DNA damages correctly, we calculated the *heterogeneity of response* [24–26] (e.g., the distribution of % DNA in the tail). When the true distribution of % DNA in the tail is normal, the discarded 10% cells would cause only 1.7% decrease in the confidence level with the same confidence interval (e.g., 95% confidence interval using 50 comets = 93.7% confidence interval using 41 comets). Thus, HiComet could provide a satisfactory performance with a sufficient number of comets in one image (e.g., 25 comets [24]).

### Characterization of comet parameters

**Fig. 10:**
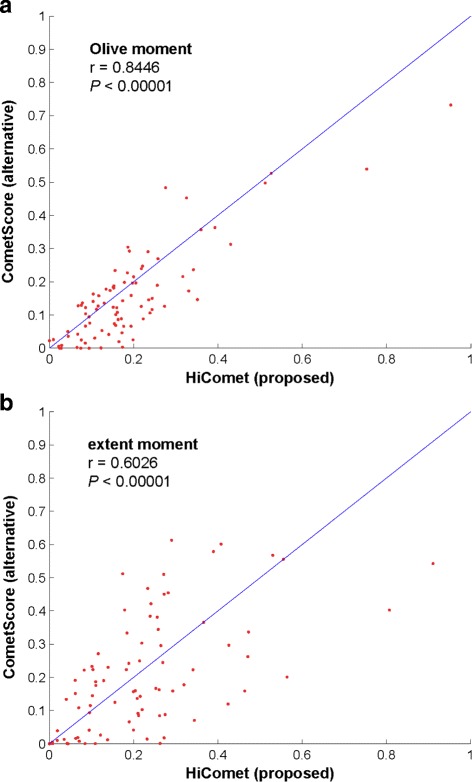
Correlation between normalized tail moments calculated by two tools: HiComet and CometScore (TriTek Corp., Sumerduck, VA). Eighty-six comets were randomly sampled from the 35 test images used. **a** Olive moment; **b** extent moment

Once a comet is recognized, it should be characterized by measuring its key parameters such as the tail moment. We compared the tail moments calculated by HiComet with those calculated by an existing program called CometScore (TriTek Corp., Sumerduck, VA). Figure 10 shows the correlation between the Olive moment and the extent moment calculated with these methods. For both the moments, the correlation was positive. The correlation for the Olive moment (0.8446) was higher than that for the extent moment (0.6026).

**Fig. 11:**
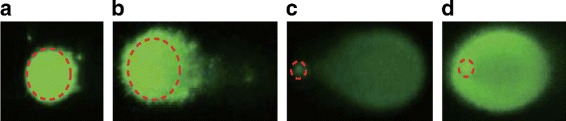
Comets and their heads. Green images represent comets, on which red circles indicate the location of the head, as manually determined by domain experts. The four images represent different states, as follows: **a** normal, **b** necrosis, and **c** and **d** apoptosis. For **a** and **b**, the head diameters are similar to the comet heights. In contrast, the head diameter is smaller than the comet heights in **c** and **d**. CometScore (TriTek Corp., Sumerduck, VA) does not consider this fact and tends to overestimate the head diameter, resulting in an underestimation of the tail moment. More details of **c** and **d** are listed in Table 3

The discrepancy was mainly due to the difference in the definition of the comet head. CometScore assumes that the diameter of the comet head is identical to the height of the comet. This assumption is reasonable for certain cases (e.g., normal and necrosis; see Fig. 11), but fails to model the comet shape in other cases (e.g., apoptosis; see Fig. 11). Consequently, CometScore tends to overestimate the diameter of the head in case of apoptosis comets, resulting in underestimation of the tail moment. This is reflected in the correlation plots in Fig. 10, where the Olive or extent moment values calculated by HiComet were higher than those calculated by CometScore. The difference was more noticeable for the extent moment than for the Olive moment. This was likely because of the difference in the definitions of the two moments. As defined in Eq. (3), the extent moment is the product of TDNA (the fraction of total DNA contained in the tail) and the tail length (the distance between the head boundary and the end of the tail). If the head size is overestimated, the calculated tail size becomes lower than the actual value. This lowers both the TDNA and the tail length values, resulting in a lower extent moment value as well.

**Table 3:** Details of the comet images shown in Fig. 11c and d

	Method	Comet	Comet	Head	Tail	Tail	TDNA	Extent	Olive
		length (px)	height (px)	diameter (px)	length (px)	dist.	(%)	moment (%)	moment (%)
Fig. 11c	HiComet	84	46	5	78	48	99.53	77.64	47.78
	CometScore	84	49	49	35	46	74.84	26.19	34.94
Fig. 11d	HiComet	82	67	13	58	28	95.60	55.46	26.77
	CometScore	100	74	74	26	33	65.00	16.92	21.07

In comparison, the Olive moment is defined as the product of TDNA and the tail distance (the distance from the CPH to the CMT). Thus, for the Olive moment, overestimating the head size lowers the TDNA value, but often increases the tail distance due to the shift in the CMT towards the end of the tail. These two affect the Olive moment calculation in opposite directions. Consequently, the underestimation of the Olive moment by CometScore tends to be less significant than that of the extent moment, and the Olive moment values estimated by CometScore show higher correlation with those estimated by HiComet than the extent moment values (*r*=0.8446 vs. *r*=0.6026 in Fig. 10a). This observation also confirmed that the Olive moment was a more robust parameter than the extent moment.

## Conclusion

We demonstrated HiComet, an automated tool for high-throughput comet-assay analysis. The key features of HiComet were described. First, HiComet automatically recognizes individual comets from the input image without making any assumptions on the number of comets or their location. This is critical for reducing the time taken for analyzing high-throughput assays with many comets. Second, HiComet can detect overlapping comets and isolate them. Without this feature, researchers would have to discard overlapping comets, even though the comets involved may be eligible for analysis. Given that overlaps occur frequently in typical high-throughput comet assays, this functionality would be useful for maintaining sufficient comet counts for analysis by salvaging parts of overlapping comets. Third, HiComet can characterize each of the recognized comets and report their key parameters such as tail moments without making overly simplified assumptions about comet shapes, as some existing tools do. Given the effectiveness of HiComet, it could greatly facilitate high-throughput comet-assay analysis by accelerating its most rate-limiting steps.
