# Correction to: HiComet: a high-throughput comet analysis tool for large-scale DNA damage assessment

**DOI:** 10.1186/s12859-018-2186-2

**Published:** 2018-05-11

**Authors:** Taehoon Lee, Sungmin Lee, Woo Young Sim, Yu Mi Jung, Sunmi Han, Joong-Ho Won, Hyeyoung Min, Sungroh Yoon

**Affiliations:** 1 Department of Electrical and Computer Engineering, Seoul National University, Seoul, 08826 Korea; 2 R&D Center, Wearable Healthcare, Gyeonggi-do, 16954 Korea; 3 Research Division, NanoEnTek, Seoul, 08389 Korea; 4 Department of Statistics, Seoul National University, Seoul, 08826 Korea; 5 College of Pharmachy, Chung-Ang University, Seoul, 06974 Korea; 6 Bioinformatics Institute, Seoul National University, Seoul, 08826 Korea

## Correction

Due to an error introduced during typesetting of this article [1], the author affiliations have been accidentally omitted in the PDF. The author affiliations can be found in this correction:

1. Department of Electrical and Computer Engineering, Seoul National University, 08826 Seoul, Korea.

2. R&D Center, Wearable Healthcare, 16954 Gyeonggi-do, Korea.

3. Research Division, NanoEnTek, 08389 Seoul, Korea.

4. Department of Statistics, Seoul National University, 08826 Seoul, Korea.

5. College of Pharmachy, Chung-Ang University, 06974, Seoul Korea.

6. Bioinformatics Institute, Seoul National University, 08826 Seoul, Korea.

## References

[1] Lee T, Lee S, Sim WY, Jung YM, Han S, Won J, Min H, Yoon S (2018). HiComet: a high-throughput comet analysis tool for large-scale DNA damage assessment. BMC Bioinformatics.

